# Examining contraceptive utilization behavior in Pakistani women

**DOI:** 10.1186/s12978-024-01815-z

**Published:** 2024-07-03

**Authors:** Lubna Naz, Umema Amin Siddiqui, Shyamkumar Sriram

**Affiliations:** 1 School of Economics and Social Sciences, Institute of Business Administration (IBA), 75270 University Road, Karachi, Pakistan; 2 Department of Social and Public Health, Ohio University, Athens, OH 45701 USA

**Keywords:** Contraceptive knowledge, Reproductive health, Sociocultural norms, Spousal age difference, Women’s intra-household bargaining power

## Abstract

**Background:**

There is a dearth of research examining the couple characteristics in determining contractive utilization behavior in developing countries. This study fills the gap by analyzing the roles of women’s intra-household bargaining power and spousal age differentials in predicting contraceptive utilization behavior in Pakistani women*.*

**Methods:**

A sample of 13,331, excluding pregnant and sexually inactive married women aged 15–49, was extracted from the Pakistan Demographic and Health Survey 2017–18. The dataset is cross-sectional. Exploratory analysis was used to examine the pattern of contraceptive knowledge, types of contraceptive utilization, and intention to use contraceptives among women. Furthermore, binary regressions were employed to examine the association of women's intrahousehold bargaining power and spousal age difference with contraceptive utilization without and after accounting for all potential covariates.

**Results:**

Only 33% of women use contraceptives, while 30% express an intention to use contraceptives in the future. Almost all women (98%) knew about modern contraceptives. Compared to same-age couples, higher odds of current contraceptive use are observed among women whose husbands are at least 20 years older than them or whose husbands are young to them. The odds of the intention to use contraceptives tend to increase with the increase in spousal age difference. Women’s intra-household bargaining is a significant predictor of current contraceptive utilization and intention to use contraceptives.

**Conclusion:**

Findings underscore the importance of considering the couple’s characteristics in reproductive healthcare programming and policies.

**Recommendation:**

Greater women's intra-household bargaining power and smaller spousal age differences are associated with higher contraceptive usage. Empowering women and promoting their decision-making authority within households can enhance reproductive health outcomes.

## Background

Globally, 287,000 maternal deaths were reported in 2020. Most of these deaths occur in low-middle-income countries (LMICs), accounting for 95% of the total [1]. The primary cause of maternal mortality is high-risk fertility behavior (HRFB) [1, 2]. Additionally, the underfinanced healthcare systems, poorly governed healthcare service delivery, and limited outreach of maternal healthcare services contribute to maternal deaths [3, 4].

Pakistan ranks fifth amongst the most populous countries in the world [5]. In the 1960s, the population growth rate of Pakistan was around 2.7% per annum. However, during the 1970s and 1980s, the Total Fertility Rate (TFR) soared to about six to seven births per woman, increasing population growth by 3% per annum [6]. These population trends have had implications for reproductive health in the country. Out of the 9 million annual pregnancies in Pakistan, a staggering 48% or 4.2 million of them are unintended. Of this unintended pregnancy rate, approximately 54% or 2.2 million cases are managed through induced abortions, highlighting the significant impact of reproductive choices in the population dynamics [cite Sathar]. According to UN projections, by the year 2050, Pakistan’s population will reach an estimated 307 million, surpassing Indonesia and becoming the fourth most populous country [7]. In Pakistan, women reveal a preference for an average of 2.9 children. However, the average number of children born to women is 3.6, resulting in 0.7 additional children either born at an undesirable time or unwanted [8].

Pakistan has spent a meager 0.8%-1.2% of its national income on health over the last six years [5], while the share of spending on reproductive, maternal, newborn, and child health sector (RMNCH) hovers around 21% of total healthcare spending [9]. In addition, it is worth noting that the expenditure on health as a percentage of GDP has been stagnant at 1.2% for the past several years [5]. The lack of access to family planning services is considered one of the leading causes of unintended births [10]. Another critical issue is the contraception gap among women; they desire more control over the timing and number of births, but they do not utilize any contraceptive method. Apart from accessibility issues in contraceptive uptake, women’s weak intrahousehold bargaining decisions prevent them from exercising fertility planning [11]. The firmly held cultural and social norms, religion, and misperceptions about family planning further limit the autonomy of women relating to reproductive health [12]. The lack of women’s autonomy in family planning decisions results in unplanned pregnancies and poor maternal and child health [13, 14]. The risk of unplanned pregnancies is exacerbated by inadequate allocation of resources to reproductive healthcare, restricting the outreach of family planning products and services, safe abortions, and nutrition programs to fewer women [15].

Moreover, in a conventional society like Pakistan, the premium is placed on the reproductive potential of women, which promotes the social acceptability of early-age marriage of girls, resulting in age heterogamy [16, 17]. In such situations, the husband is generally older than the wife. Age heterogamy is associated with a lower adoption to birth spacing and more unintended pregnancies among women [17]. More specifically, an older-wife to a younger-husband difference (or a negative difference) has been found to positively affect the likelihood of contraceptive utilization, particularly among young women. In contrast, an older-husband to a younger-wife difference (or a positive difference) is negatively associated with the probability of using current contraceptives [18]. In essence, the age difference in marriages can influence the use of contraceptives, with a positive effect when the wife is older and a negative effect when the husband is older. Longitudinal studies established that reducing the ratio of unplanned pregnancies can effectively translate into improved academic records, increased labor productivity, a higher labor force participation rate, better health prospects, and reduced crime rates in society [19].

Several studies have been conducted on fertility planning; however, there remains a compelling need for more rigorous research to understand how the characteristics of couples shape their contraceptive behavior. This study aims to bridge this gap by delving into how women’s intra-household bargaining power and spousal age difference interplay with the contraceptive behavior of married women in Pakistan. The study utilizes the data from the Pakistan Demographic and Health Survey 2017–18, a nationally representative dataset. Various measures, such as contraceptive use, knowledge of different contraceptive methods, and women’s intention toward future contraceptive use, are employed to assess contraceptive behavior, unlike previous studies that transformed women’s decision-making scores into a bounded variable, disregarding the fact that women's autonomy is a multifaceted concept that cannot be categorized as “existing” or “not existing [20]. This study treats women’s decision autonomy as a continuous variable [21].

Moreover, the study examines the spousal age difference by calculating the husband-wife age difference at their first marriage. The outcomes are mainly organized into three categories: no age difference, negative age-difference, and positive age difference. The exploratory data analysis is used to address the research objective. The research findings offer deep insight into the pivotal role of a couple's characteristics in reproductive health decisions.

## Methods

### Data and sample

This study employed the Pakistan Demographic and Health Survey (PDHS). It is a nationally representative dataset. The PDHS gathers data on the participants' socio-demographic, economic, and health characteristics, mainly married women aged 15–49. This study extracted samples of 13,331 married women after excluding currently pregnant women (*n* = 1733) and those who were never sexually active (*n* = 4).

### Outcome variables

The PDHS provides data on contraception behavior in married women aged 15–49. Detailed information is given on various types of contraceptive methods, including women use pills, condoms, injectables, sterilization, intra-uterine devices (IUDs), implants, emergency contraception (EC), lactational amenorrhea method (LAM), or standard days method (SDM), which are known modern contraceptive methods, and abstinence or withdrawal, termed as traditional contraceptives [11, 22]. The dataset also contains information on contraceptive knowledge about various methods and details on the future intention of contraceptive use among women. This study employed data on family planning to assess the contraceptive behavior of married women aged 15–49. Various indicators of contraceptive behavior were used. The contraceptive use was used as a binary variable, where '1' denotes traditional and modern contraception, and '0' implies non-use. The “non-use” refers to women who had no intention of using contraceptives at all and those who desired to use contraceptives later but not presently. Contraceptive knowledge was used as a categorical variable, comprising three responses as follows, “knows no method,” “knows only traditional method,” and “knows only modern method.” For analyzing the future intention to use contraceptives, the study used a binary variable (1/0), where 1 means “intention to use contraceptive in future,” and 0 implies “no such intention.”

### Women’s bargaining power

Women’s Bargaining Power Index (WBPI) was constructed using 13 indicators, encompassing women’s decision-making autonomy in areas such as contraceptive use, allocation of household income, spending decisions, healthcare choices, big household purchases, and property ownership. Additionally, the index included information on women’s attitudes towards justifications for spousal beating. In rural contexts, land ownership holds particular significance, as it tends to confer financial security and empowerment to women, leading to increased control over their lives and active participation in family decision-making processes [23].

Multiple Correspondence Analysis (MCA) was employed using several sequential steps. Initially, we converted variables with multiple responses into a dichotomous variable, where “1” indicated the respondent herself or jointly with her husband, and “2” denoted others. Subsequently, the Cronbach’s Alpha coefficient was calculated to assess internal consistency, yielding a value of 0.79. Following this, MCA was utilized to construct the index. Lastly, normalization of the Women’s Bargaining Power Index (WBI) was performed to obtain standardized scores. A similar methodology has been widely adopted in the existing literature to quantify women's autonomy [21].

### Spousal age difference

The spousal age difference was measured by calculating the age difference between spouses, precisely husband age minus wife age in years. To create a categorical variable, we organized the data into six distinct age-difference groups as follows: 1) same-age couple, 2) husband is younger than wife, 3) husband is 1–5 years older, 4) husband is 6–10 years older, 5) husband is 11–20 years older, 6) husband is over 20 years older.

### Other covariates

The study employed a few covariates. The age of women was divided into three groups: ‘15–24 years’, ‘25–34 years’, and ‘35–49 years. The education level of both women and their husbands was recoded as 'no education,' 'primary education,' 'secondary education', and 'higher education’. The socioeconomic status of households was classified into five tiers: 'poorest,' 'poor,' 'middle', 'rich,' and 'richest.' Place of residence was taken as a dichotomous variable, with '1' denoting rural and '0' representing urban areas. The number of living children was a categorical variable, encompassing the following options, 'no children,' '1–2 children', '3–4 children', '5–6 children', and 'more than 6 children'. Women’s desire for additional children was coded as ‘1’ if they did not wish to have another child and '0' otherwise. The exposure to electronic media was gauged through TV and newspaper usage, coded as ‘1’ if the woman had watched TV at least once a week/ read a newspaper at least once a week”, and ‘0’ otherwise. A ‘bank account’ and ‘mobile phone’ ownership were measured as binary variables, with ‘1’ representing ownership and '0' indicating otherwise. A woman's employment status was a categorical variable, with ‘1’ if they had worked in the past 12 months and '2’ if they had not.

### Statistical analysis

We used multivariate logistic regression to analyze the association of women’s autonomy and spousal age difference with contraceptive utilization. Two binary outcome variables were used: current contraceptive use and intention to use contraceptives. The following two models were estimated for each outcome variable. Model-1 featured women’s bargaining power and spousal age difference as only explanatory variables, whereas Model-2 accounted for all potential covariates along with WBPI and spousal age difference, Tables 2 and 3.

## Results

Table 1 presents the distribution of various indicators of contraception behavior among married women. The current contraceptive use was reported by 35% of women. Nearly all women (98%) had knowledge of modern contraceptives. Conversely, a mere 1.6% had no knowledge about any method. Among women not currently using contraceptives, including pregnant women, 30.3% expressed intent to use contraceptives in the future, while 69.7% did not show any inclination toward contraceptive utilization.

**Table 1:** Contraceptive behavior of married women aged 14–49

Measures	N	Percent	lower	Upper
Contraceptive use				
Yes	4,753	35.6	34.8	36.4
No	8,578	64.3	63.5	65.1
Contraceptive knowledge				
No knowledge about any method	222	1.6	1.4	1.8
Traditional method	25	0.18	0.12	0.27
Modern method	13, 084	98.1	97.9	98.3
Current contraceptive method				
Not using	8,578	64.3	63.5	65.1
Flokloric	16	0.12	0.07	0.19
Traditional	1,274	9.5	9.0	10.0
Modern	3,463	25.9	25.2	26.7
Intention to use				
Yes	3,111	30.3	29.3	31.1
No	7, 191	69.7	68.8	69.9

Source: PDHS 2017–18

Table 2 presents the characteristics of participants in the study. The women’s bargaining power index exhibited an average value of 3.5. The spousal age difference revealed that most husbands (45%) were 1–5 years older than their wives, while a smaller percentage (6%) were younger, and 8% constituted couples of the same age. Among the distribution of married women within different age groups, 20% fell within 15–24 years, 41% were in the 25–34 age range, and 39% belonged to the 35–49 age group. Educational attainment statistics indicated that 50% of women had not received formal education, approximately 20% had completed secondary education, and a smaller proportion of 15% had achieved higher education. Conversely, husbands were observed to have higher levels of education than their female spouses: 35% of husbands had possessed secondary education, 19% had higher qualifications, and 29% had not obtained any formal education.

**Table 2:** Characteristics of married women aged 15–49 (*n* = 13,331)

Variables	Mean/Proportion	CI at 95% upper	CI at 95% lower
WBPI	3.51	3.42	3.63
Spousal age difference			
Same age couple	0.06	0.05	0.06
Husband is younger to wife	0.08	0.07	0.08
Husband is 1–5 years older than wife	0.45	0.44	0.46
Husband is 6–10 years	0.27	0.26	0.27
Husband is 11–20 years	0.11	0.10	0.12
Husband is over 20 years older	0.02	0.01	0.02
Women’s education			
No education	0.50	0.49	0.51
Primary	0.14	0.13	0.15
Secondary	0.20	0.19	0.21
Higher	0.15	0.13	0.15
Women’s age			
15–24	0.20	0.19	0.21
25–34	0.40	0.39	0.41
35–39	0.39	0.38	0.40
Women’s say in choosing husband	0.80	0.79	0.81
Yes	0.80	0.79	0.82
No	0.19	0.17	0.20
Women worked in last 12 months			
Yes	0.14	0.13	0.14
No	0.86	0.85	0.87
Women’s own mobile phone			
Yes	0.39	0.58	0.63
No	0.60	0.36	0.41
Women’s own bank account			
Yes	0.06	0.05	0.06
No	0.94	0.93	0.95
Husband’s education			
No education	0.29	0.27	0.31
Primary	0.15	0.14	0.16
Secondary	0.35	0.33	0.37
Higher	0.19	0.17	0.21
Women reading newspaper			
Not at all	0.85	0.84	0.87
At least once a week	0.14	0.12	0.15
Women watching television			
Not at all	0.37	0.34	0.40
At least once a week	0.62	0.59	0.65
Place of residence			
Rural	0.63	0.60	0.65
Urban	0.36	0.60	0.59
Household wealth status			
Poorest	0.18	0.15	0.21
Poorer	0.19	0.17	0.21
Middle	0.20	0.18	0.22
Richer	0.20	0.18	0.23
Richest	0.20	0.18	0.23
Women want more children			
Yes	0.53	0.51	0.54
No	0.46	0.45	0.48
Number of living children			
0	0.14	0.13	0.15
1–2	0.30	0.29	0.32
3–4	0.31	0.29	0.32
5–6	0.16	0.15	0.17
6 +	0.07	0.06	0.08

Authors calculations, DHS 2018–19

The data on women’s employment highlights that merely 13% of women worked during the 12 months before the survey. A significant majority of women (63%) resided in rural areas. When examining exposure to information technology, it was discovered that 39% of women owned mobile phones. However, it is important to note that owning a mobile did not necessarily translate to the freedom to utilize it, given the constraints imposed by a patriarchal society and limited resources. The accessibility to banking services was restricted to a mere 6% of women.

Regarding media exposure, 14% of women reported reading newspapers at least once a week, while 60% had access to and watched television. About 80% of women reported having a say in selecting their life partner. On average, each woman had more than three living children, with 53% expressing a desire for an additional child.

**Fig. 1:**
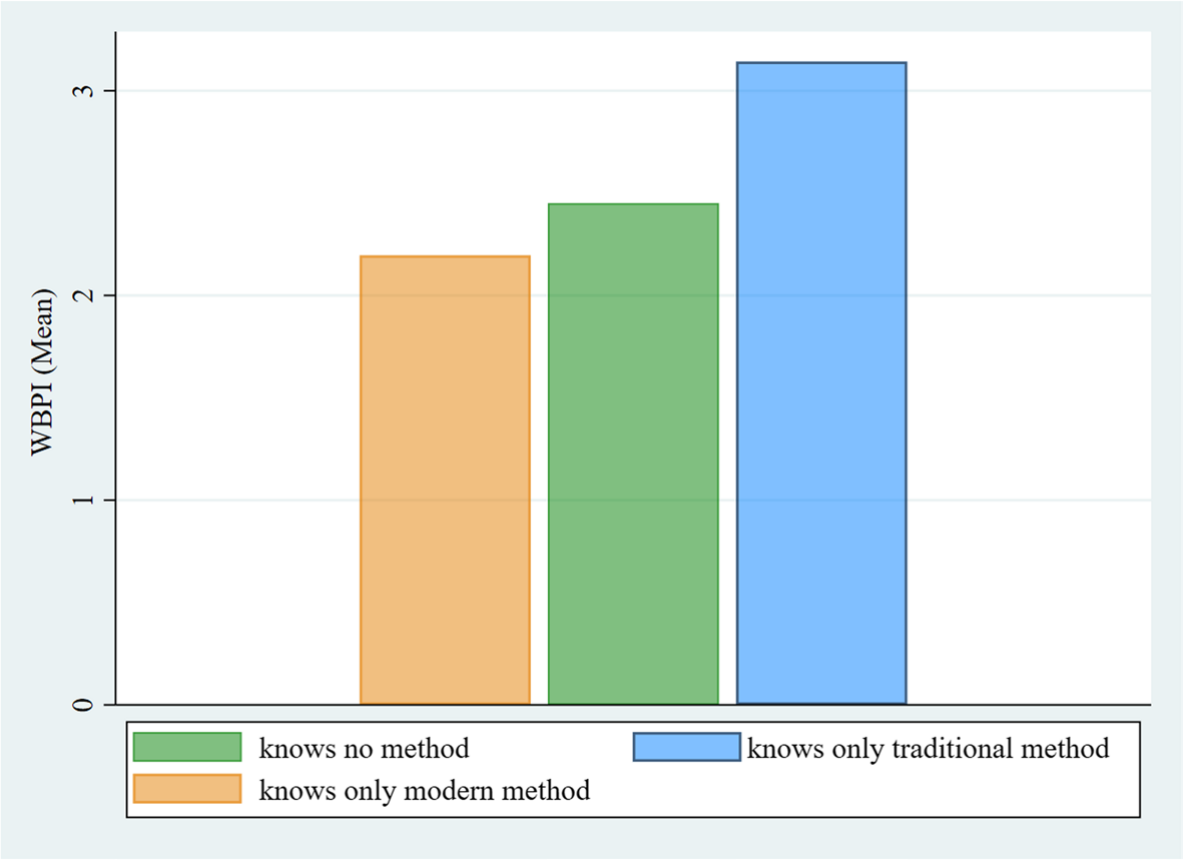
Knowledge of contraceptive methods among women with different WBPI mean values (n=13,331) in 2017-18 in Pakistan

Figure 1 illustrates the pattern of contraceptive knowledge by WBPI. The highest mean WBPI score aligned with the knowledge limited to traditional methods, while a lack of knowledge regarding any contraceptive method was predominantly observed among women with comparatively lower average WBPI values. Notably, women with the lowest WBPI scores knew only modern contraceptives.

**Fig. 2:**
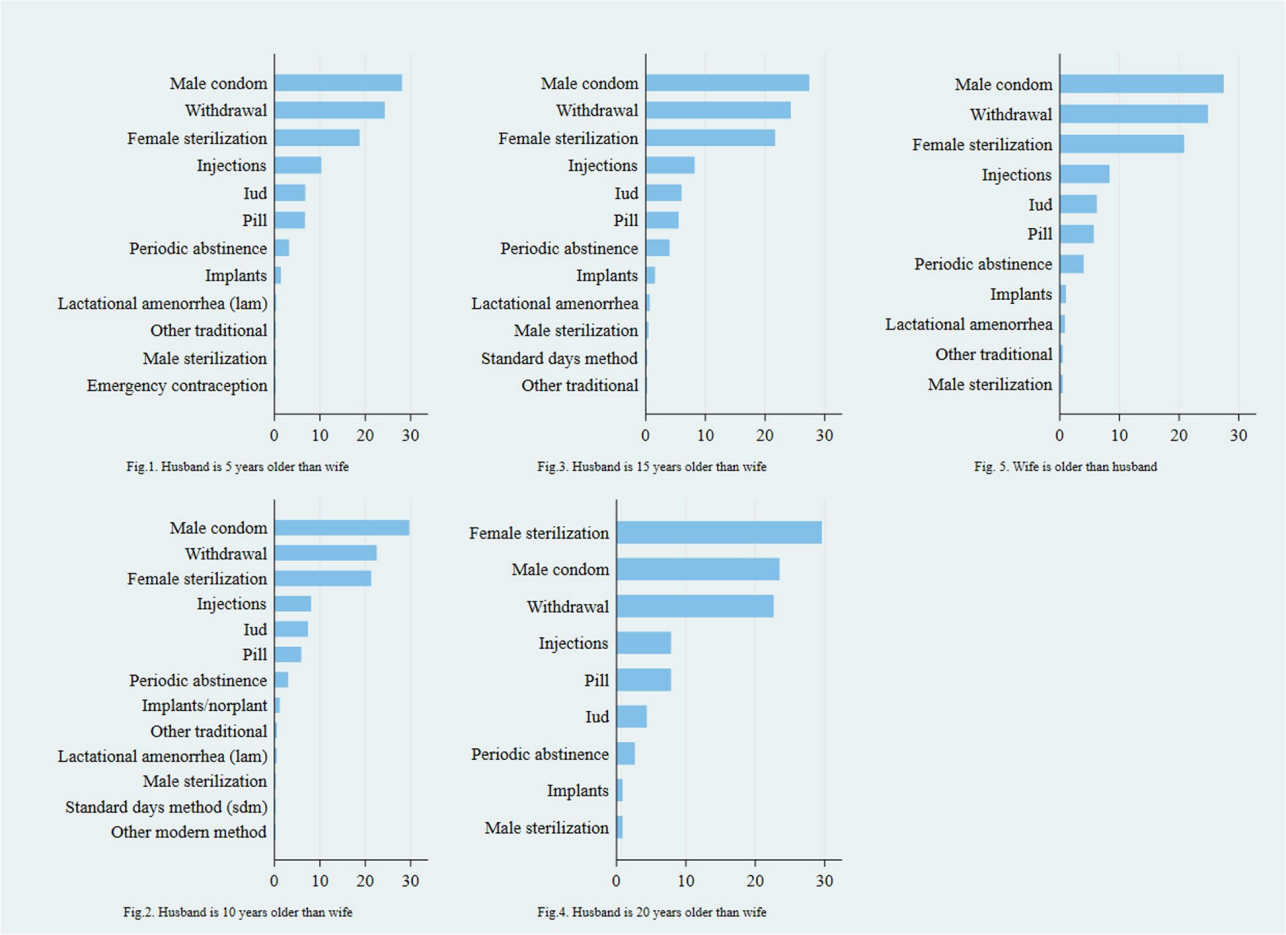
The current contraceptive use among married women across husband-wife age differentials in Pakistan in 2017-18

Figure 2 depicts the pattern of methods of contraceptive use associated with the spousal age difference. The couples of the same age utilized nearly all birth spacing methods. However, adopting long-term methods, such as male sterilization, by male spouses was less prevalent than short-term methods. Women exhibited a minimal preference for long-term modern birth control methods (i.e., implants) across most positive husband-wife age differentials, except when the husband was over twenty years older than his wife. When the husband-and-wife age ranged from 1 to 20 years, higher male condom and withdrawal rates were observed. A similar pattern was observed when a wife was older than her husband. The uptake of short-term family planning methods, including IUDs, pills, periodic abstinence, and injections, was more commonly observed across all age differentials, even when a reverse age gap existed between the husband and wife. Another noteworthy pattern in contraceptive use was the relatively higher male participation in contraceptive uptake when the husband was either 15 or 20 years older than his wife or when the wife was older. Nevertheless, males' uptake of long-term contraceptives was insignificant across all age-differential groups.

The findings from the Model-1 model revealed that women’s bargaining power index (WBPI) had a significant and positive association with the odds of using contraceptives, see Table 3. Each additional unit of WBPI corresponded to a 1.19-fold increase in the odds of using contraception [OR = 1.19, 95% CI: 1.16–1.23]. Regarding spousal age difference, considering no age difference as a base category, husbands who were younger than their wives and those who were over 20 years older than them had 25% and 32% lower odds of contraceptive use [OR = 0.75, 95% CI: 0.61–0.91; OR = 0.68, 95% CI: 0.49–0.69], respectively. However, the results for other spousal age differentials did not yield significant associations.

**Table 3:** Logistic regression of current contraceptive use among married women aged 15–49

Variables	β	$SE.(\beta )$	Odd ratio (95% CI)	β	$SE.(\beta )$	Odd ratio (95% CI)
Outcome variable: Current contraceptive uptake						
WBPI (normalized)	0.18	0.013	1.19***(1.16–1.23)	0.04	(0.0174)	1.05***(1.01–1.08)
Women’s education (RC: No education)						
Primary				0.26	(0.0675)	1.29***(1.13–1.48)
Secondary				0.42	(0.0698)	1.53***(1.33–1.75)
Higher				0.71	(0.0921)	2.03***(1.69–2.43)
Women’s age (RC: 15–24 years)						
25–34 years				-0.20	(0.0746)	0.81***(0.70–0.94)
35–49 years				-0.83	(0.0849)	0.43***(0.36–0.51)
Women worked in the past 12 months (RC: No)						
Yes				-0.14	(0.0623)	1.15**(1.02–1.30)
Women’s say in choosing husband (RC: No)						
Yes				0.086	(0.0539)	1.09(0.98–1.21)
Women have mobile phones (RC: No)						
Yes				-0.004	(0.0491)	0.99(0.90–1.09)
Women have bank account (RC: No)						
Yes				-0.22	(0.0907)	0.79**(0.66–0.95)
Husband’s education (RC: No education)						
Primary				0.26	(0.0675)	1.14*(0.99–1.31)
Secondary				0.42	(0.0698)	0.97(0.86–1.10)
Higher				0.71	(0.0921)	1.04(0.90–1.19)
Reading newspaper (RC: Not at all)						
At least once a week				0.053	(0.0648)	1.05(0.92–1.05)
Watching TV (RC: Not at all)						
At least oonce a week				0.28	(0.0510)	1.32***(1.20–1.46)
Age heterogamy (RC: No age difference between couple)						
Husband is younger to wife	-0.28	0.09	0.75**(0.61–0.91)	0.01	(0.120)	1.01(0.80–1.28)
1–5 years older than wife	0.0005	0.078	1.01(0.85–1.16)	-0.05	(0.0950)	0.94(0.78–1.13)
6–10 years	0.12	0.081	1.13(0.97–1.32)	-0.04	(0.0982)	0.95(0.79–1.16)
11–20 years	0.03	0.091	1.03(0.86–1.23)	-0.13	(0.110)	0.87(0.70–1.08)
More than 20 years	-0.37	0.166	0.68**(0.49–0.94)	-0.36	(0.195)	0.69*(0.47–1.02)
Women want more children (RC: Yes)						
No				1.25	(0.0536)	3.5***(3.15–3.89)
Number of living children (RC:None)						
1–2				3.39	(0.241)	29.9***(18.6–47.9)
3–4				4.07	(0.244)	1.93***(1.65–2.26)
5–6				4.37	(0.248)	2.13***(1.79–2.53)
6 +				4.28	(0.255)	2.26***(1.86–2.73)
Wealth index (RC: Poorest)						
Poorer				0.41	(0.0727)	1.50***(1.30–1.73)
Middle				0.66	(0.0795)	1.93***(1.65–2.26)
Richer				0.75	(0.0875)	2.13***(1.79–2.53)
Richest				0.81	(0.0974)	2.26***(1.86–2.73)
Place of residence (RC: Urban)						
Rural				-0.04	(0.0488)	0.95(0.86–1.04)
Constant	-1.23	0.088	0.29***(0.24–0.34)	-5.52	(0.283)	0.003***(0.002–0.005)
Observations		13,331	13,331			12,671
Wald statistic			211			1975
Prob > chi2			0.000			0.00

^*^RC means reference category

^**^Robust standard errors in parentheses *** *p* < 0.01, ** *p* < 0.05, * *p* < 0.1

In Model-2, the WBPI was positively associated with contraceptive use, whereas the relationship between the spousal age difference and the probability of contraceptive use was insignificant. After accounting for confounding variables, the odds attributed to WBPI [OR = 1.05, 95% CI: 1.01–1.08] were slightly attenuated, see Table 3. Nonetheless, WBPI persisted as a significant predictor of the likelihood of contraceptive use among women in both models, underscoring the pivotal role of WBP in contraceptive use in Pakistan. Meanwhile, the spousal age difference was insignificant when other covariates were considered.

Among other covariates, as women’s educational attainment improved – compared to those women with no education – primary, secondary, and higher education showed an increased odd ratio of using contraceptives, with values of 1.29, 1.53, and 2.09, respectively, Table 3.

Women’s age was inversely but significantly associated with the likelihood of using contraception. In comparison to the reference category, women aged 15–24 years, those in the age groups of 25–34 years and 35–49 showed lower probabilities of contraceptive use by 42% [OR = 0.81, 95% CI: 0.70–0.94] and 57% [OR = 0.43, 95% CI: 0.36–0.51], respectively. This trend might be attributed to women approaching a less fertile age; as they age, the inclination to conceive diminishes, resulting in reduced motivation for contraceptive use [24]. Women employed within the last 12 months demonstrated 1.15 times higher odds [OR = 1.15, 95% CI: 1.02–1.30] of utilizing contraceptives compared to those without employment. Similarly, women with varying numbers of children, particularly those with six or more children, exhibited higher odds of using contraceptives compared to women who did not have children.

Notably, there was an insignificant association between the husband’s education and the likelihood of contraceptive utilization at the 95% confidence interval. Women who maintained a bank account had a 21% lower probability [OR = 0.79, 95% CI: 0.66–0.95] for current contraceptive use. Additionally, a positive and significant relationship was observed between exposure to media and contraceptive use; women who had watched TV at least once a week showed 1.32 times odds (OR = 1.32, 95% CI: 1.20–1.46] of using current contraceptives.

Table 4 shows the logistic regression results of the intention to use contraceptives. In the Model-1 model, WBPI was positively associated with the odds of future contraceptive use intention [OR = 1.09, 95% CI: 1.05–1.12]. Spousal age difference exhibited a negative association with the contraceptive use intention among women at the 95% confidence level. Compared to the same age couple, all age difference groups were associated with lower odds of future contraceptive use intention, as follows: age difference 1–5 years [OR = 0.57, 95% CI: 0.44–0.73], 6–10 years [OR = 0.77, 95% CI: 0.63–0.94], 11–20 years [OR = 0.78, 95% CI: 0.63–0.95], and over 20 years [OR = 0.52. 95% CI: 0.41–0.68], and negative age difference [OR = 0.34, 95% CI: 0.21–0.54].

**Table 4:** Logistic regression of the contraceptive use intention for the future among married women aged 15–49

	β	$SE.(\beta )$	Odd ratio (95% CI)	β	$SE.(\beta )$	Odd ratio (95% CI)
WBPCI (normalized)	0.087	0.017	1.09*** (1.05–1.12)	0.0657	(0.0225)	1.06*** (1.02–1.11)
Women’s education (RC: No education)						
Primary				0.460	(0.0856)	1.58***(1.02–1.11)
Secondary				0.761	(0.0855)	2.14*** (1.81–2.53)
Higher				0.866	(0.116)	2.37***(1.89–2.98)
Women’s age (RC: 15–24 years)						
25–34 years				-0.351	(0.0717)	0.70*** (0.61–0.81)
35–49 years				-1.365	(0.0961)	0.25*** (0.21–0.30)
Women worked in the past 12 months (RC: No)						
Yes				0.0608	(0.0821)	1.06 (0.90–1.24)
Women’s say in choosing husband (RC: No)						
Yes				0.318	(0.0712)	1.37***(1.19–1.58)
Women have mobile phone (RC: No)						
Yes				0.187	(0.0633)	1.18***(1.06–1.36)
Women have bank account (RC: No)						
Yes				0.152	(0.112)	1.16 (0.93–1.44)
Husband’s education (RC: No education)						
Primary				0.205	(0.0886)	1.22**(1.03–1.46)
Secondary				0.191	(0.0742)	1.21**(1.04–1.39)
Higher				-0.0281	(0.0916)	0.97(0.81–1.16)
Reading newspaper (RC: Not at all)						
At least once a week				0.168	(0.0796)	1.18**(1.01–1.38)
Watching TV (RC: Not at all)						
At least once a week				0.217	(0.0647)	1.24*** (1.09–1.41)
Spousal age difference (RC: No age difference between couple)						
Husband is younger to wife	-0.559	0.126	0.57*** (0.44–0.73)	-0.106	(0.140)	0.89(0.68–1.18)
1–5 years older than wife	-0.253	0.100	0.77***(0.63–0.94)	-0.326	(0.108)	0.72***(0.58–0.89)
6–10 years	-0.249	0.104	0.78*** (0.63–0.95)	-0.335	(0.113)	0.71***(0.57–0.89)
11–20 years	-0.643	0.123	0.52*** (0.41–0.68)	-0.574	(0.133)	0.56***(0.43–0.73)
More than 20 years	-1.075	0.239	0.34*** (0.21–0.54)	-0.801	(0.247)	0.44***(0.27–0.78)
Women want more children (RC: Yes)						
No				-0.0923	(0.0733)	0.911(0.78–1.05)
Number of living children (RC: None)						
1–2				0.467	(0.0767)	1.5***(1.37–1.85)
3–4				0.751	(0.0925)	2.1***(1.76–2.84)
5–6				1.071	(0.114)	2.9***(2.33–3.64)
6 +				1.275	(0.143)	3.5***(2.70–4.73)
Wealth index (RC: Poorest)						
Poorer				0.0128	(0.0820)	1.01(0.86–1.18)
Middle				-0.0242	(0.0969)	0.97(0.80–1.80)
Richer				-0.161	(0.108)	0.85(0.68–1.05)
Richest				-0.421	(0.122)	0.65***(0.51–0.83)
Place of residence (RC: Urban)						
Rural				0.174	(0.0623)	1.18***(1.05–1.34)
Constant	1.024	0.112	0.35***(0.28–0.44)	-1.737	(0.165)	0.176***(0.12–0.24)
Observations			8,574			7,987
Wald statistic			75.5			618
Prob > chi2			0.000			0.00

^*^RC means reference category

^**^Robust standard errors in parentheses *** *p* < 0.01, ** *p* < 0.05, * *p* < 0.1

The Model-2 showed a statistically significant association with both primary variables. However, the magnitude of the effect of the WBPI [OR = 1.06, 95% CI: 1.02–1.11] was slightly lower when other potential covariates were accounted for in the model. The likelihood estimates for the contraceptive use intention for an age difference of 1–5 years decreased from 0.43 (Model 1) to 0.11(Model 2) [OR = 0.89, 95% CI: 0.68–1.18]. Conversely, the odds increased marginally from 22% (see Model) to 28% (Model 2) for 6–10 years [OR = 0.72, 95% CI: 0.58–0.89], see Table 4.

Apart from the richest, all wealth groups did not show a significant association with the probability of the intention of contraceptive use when compared to the poorest. Women in rural areas were positively associated with the odds of using contraceptive use intendedness [OR = 1.18, 95% CI: 1.05–1.34] compared to their urban counterparts. This could be attributed to optimistic expectations among women regarding improved contraceptive accessibility soon. Relative to husbands without formal education, those with primary and higher qualifications, excluding higher secondary, demonstrated increased odds of intended contraceptive use. Similarly, women with higher educational attainment showed a positive association with the odds of contraceptive use intention. Unlike women’s education, the age of women was found to have a significant but negative relationship with the likelihood of using contraceptives in the future. Compared to women aged 15–24, those aged 25–34 and 36–49 had lower odds for contraceptive use intention by 30% and 75% [OR = 0.70, 95% CI: 0.61–0.81; OR = 0.25, 95% CI: 0.21–0.30], respectively.

Women who had a say in selecting their husbands at the time of their first marriage exhibited 1.37 times the odds [OR = 1.37, 95% CI: 1.19–1.58] of intending to use contraceptives compared to those who did not make such choices. Possessing a mobile phone, women’s exposure to media like TV and newspapers, as well as the number of living children, demonstrated a positive association with the likelihood of intending to use contraceptives.

## Discussion

This study analyzed contraception behavior among married aged 15–49 who were not pregnant and sexually active during 2017–18. The analysis reveals that a mere 33% of the sexually active women used current contraceptive methods. The notably low prevalence of contraceptive usage among Pakistani women can be attributed to the predominantly male-oriented societal structure, where decisions regarding contraceptive use typically lie in the hands of husbands. Another hindrance to contraceptive uptake is the profoundly ingrained perception within many households that fertility is divinely ordained, and utilizing family planning methods could be seen as opposing the will of God [23]. Research conducted in South Asia suggests that utilization of contraceptives or the practice of birth spacing is not widespread among newlywed couples, consequently contributing to higher fertility rates [11].

Another significant finding of this study is the woman's bargaining power association with the likelihood of using current contraceptives. Similarly, prior evidence suggests that the increased role of women in household decision-making contributes to birth-spacing and enhanced access to sexual and reproductive healthcare services [25].

Our results showed a higher prevalence of spousal age difference in Pakistan, as indicated by Table 2. However, the adverse effect of spousal age difference on family planning can be mitigated through women’s education [26], wealth enhancement [27], and the creation of job opportunities for women [28]. The study revealed a decreased likelihood of using current contraceptives among women with younger husbands or with husbands over 20 years older than their wives. In low-middle-income countries (LMICs), the substantial positive or negative difference in age between spouses is typically the outcomes of marriages negotiated based on factors such as dowery or bride exchange (‘give-take’) rather than the compatibility of the couple [29]. In such scenarios, couples often face substantial familial pressure to prove fertility by producing a child in order to strengthen their marital union.

The findings demonstrated a notable trend regarding women's age and contraceptive behavior; as women’s age increases, their likelihood of using contraceptives decreases. Women aged over 40 years tend to have lower fecundity and take longer to conceive than younger women. Additionally, women entering menopause can no longer conceive, potentially leading them to perceive a reduced need for contraception use [30, 31]. Conversely, some studies suggest that as women approach the later stages of their reproductive years, they are more inclined to use contraceptives to avert unintended pregnancies [32]. This could be attributed to heightened awareness of risks associated with pregnancies in later life; furthermore, when women over 40 conceive, they face an increased risk of developing obstetric complications, such as hypertension and gestational diabetes, which can pose severe threats to maternal health. Therefore, contraception is encouraged to safeguard older women against such risks [30]. In addition to that, as women age and achieve their desired family size, they may choose to limit childbearing to focus on other aspects of life, such as personal goals, career advancement, or the well-being of their family. This suggests that achieving a desired number of children prompts older women to adopt family planning measures.

Our study underscores the positive influence of women’s education on the likelihood of contraceptive behavior. Education provides women with valuable information and knowledge, empowering them to make informed decisions regarding their reproductive and sexual health [33, 34]. A highly qualified woman has greater knowledge and access to contraceptives and practices birth spacing. She is cognizant of the requirements of improving children’s quality of life rather than giving birth to many children and risking their future [26]. Furthermore, educated women allocate more time and resources to income-generating activities [35]. Moreover, educated women often adopt progressive attitudes toward reproductive health and rights. Across countries like India, Pakistan, Bangladesh, and Nepal, educated women have demonstrated improved access to healthcare services, including family planning services, which can facilitate their adoption of contraception [36]. Our findings also indicate that the probability of contraceptive use increases with the number of living children a woman has. This can be attributed to increased awareness of health risks associated with frequent childbirth among women who have experienced multiple pregnancies. This accumulated knowledge about birth-related risks prompts them to choose contraception to safeguard their health and well-being. These results align with prior research conducted in India, Nepal, and Bangladesh [37], Uganda [38], and Ethiopia [39].

In our study, contraceptive use is positively associated with household wealth. The results are consistent with existing evidence, indicating that they tend to use more contraceptives as household wealth increases. This association can be attributed to the fact that as households become more affluent, they gain greater access to resources such as education, healthcare services, and information [40]. Additionally, higher income often translates to increased purchasing power, facilitating the affordability of contraceptive methods. Furthermore, women in wealthier households might have more control over their health decisions, granting them greater autonomy in choosing whether and when to choose contraception [41]. Some studies have incorporated measures such as land and livestock ownership into the definition of wealth, and these studies have reported similar findings, suggesting a positive relationship between wealth status and contraceptive utilization [42].

Our results showed that women exposed to watching TV at least once a week are more likely to use contraceptives. This is because the media can educate women about different types of contraception, their benefits, risks, and how to access and use them effectively [36]. The results align with previous studies conducted in India, Nepal, Pakistan, and Sri Lanka affirm the positive impact of media exposure on contraceptive use [43].

In regions like Nepal, India, and Pakistan, cultural preferences for male offspring influence family planning decisions. This can lead to a desire for large families and a reluctance to use contraceptives, as having more children increases the likelihood of having a son [6, 44]. Women avoid using contraceptives until they have at least two living sons and one daughter [6]. Economic constraints, limited education opportunities, and inadequate awareness of contraceptive usage are more prevalent in rural settings, contributing to lower usage rates [36, 45].

## Conclusions

This study offers insight into crucial determinants of contraceptive behavior among married women in Pakistan. The findings emphasize the importance of women's intra-household bargaining power (WBI) and spousal age difference in shaping contraceptive behavior. Women with a stronger voice in decision-making are more likely to prioritize their reproductive health and access contraception. Therefore, strategies enhancing women's autonomy and decision-making power within households can increase contraceptive use. Furthermore, couples with significant age disparities, mainly when husbands are either younger or considerably older than their wives, exhibit reduced odds of contraceptive use. This underscores the need to address sociocultural norms and dynamics that may influence contraceptive decisions. Policymakers should, therefore, direct efforts towards promoting gender equality, ensuring accessibility of accurate information, and collaborating with community leaders and influencers to promote positive attitude towards family planning. Regardless of age difference, behavioral interventions that promote open communication and shared decision-making between partners can contribute to more effective family planning outcomes. Exposure to mass media is closely linked with higher levels of knowledge, intention, and adoption of family planning measures. Strategically using electronic and print media can result in the achievement of United Nation SDG-3 of improving good health.

## Data Availability

This article is based on secondary data obtained from the DHS program website. Prior permission was obtained from ICF to download the data and used it in the study. https://dhsprogram.com/data/available-datasets.cfm. The data is in the open domain. Anyone can access the data and replicate the results with prior permission of the DHS program-ICF.
